# One step surgical scene restoration for robot assisted minimally invasive surgery

**DOI:** 10.1038/s41598-022-26647-4

**Published:** 2023-02-22

**Authors:** Shahnewaz Ali, Yaqub Jonmohamadi, Davide Fontanarosa, Ross Crawford, Ajay K. Pandey

**Affiliations:** 1 School of Electrical Engineering and Robotics, Faculty of Engineering, Queensland University of Technology (QUT), Gardens Point, Brisbane, QLD 4001 Australia; 2 School of Clinical Sciences, Faculty of Health, Queensland University of Technology (QUT), Gardens Point, Brisbane, QLD 4001 Australia; 3 School of Mechanical, Medical and Process Engineering, Faculty of Engineering, Queensland University of Technology (QUT), Gardens Point, Brisbane, QLD 4001 Australia

**Keywords:** Biomedical engineering, Computational biophysics

## Abstract

Minimally invasive surgery (MIS) offers several advantages to patients including minimum blood loss and quick recovery time. However, lack of tactile or haptic feedback and poor visualization of the surgical site often result in some unintentional tissue damage. Visualization aspects further limits the collection of imaged frame contextual details, therefore the utility of computational methods such as tracking of tissue and tools, scene segmentation, and depth estimation are of paramount interest. Here, we discuss an online preprocessing framework that overcomes routinely encountered visualization challenges associated with the MIS. We resolve three pivotal surgical scene reconstruction tasks in a single step; namely, (i) denoise, (ii) deblur, and (iii) color correction. Our proposed method provides a latent clean and sharp image in the standard RGB color space from its noisy, blurred, and raw inputs in a single preprocessing step (end-to-end in one step). The proposed approach is compared against current state-of-the-art methods that perform each of the image restoration tasks separately. Results from knee arthroscopy show that our method outperforms existing solutions in tackling high-level vision tasks at a significantly reduced computation time.

## Introduction

Minimally invasive surgery (MIS) requires the use of a camera and a lighting source to visualize internal anatomic conditions through small incisions and the ability to correctly visualize internal anatomy in full detail is critical to the overall success of such surgical procedures. With the advancement in robotics, robot-assisted MIS (RMIS) is gaining traction where visual information obtained from an endoscope can guide the surgical procedures using automatic tissue and operating tool tracking, tissue segmentation for context and situational awareness, camera pose estimation, and reconstruction of the three-dimensional structure of the surgical site^1–3^. All these high-level tasks can get compromised by the poor quality of frames as a degraded frame can significantly limit visual and tissue specific contextual information. For instance, blurred and noisy observations may show compromised features, textures, and regions of interest. Such frames are usually discarded from clinical decisions but, in RMIS, these degraded frames could result in the failure of the whole vision-based task^4^.

**Figure 1:**
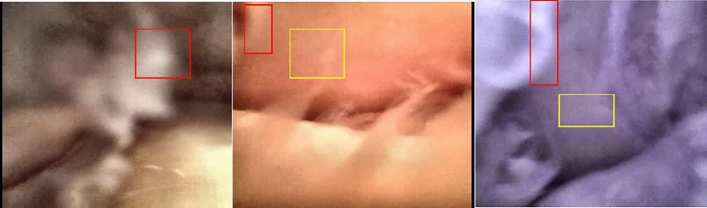
Frames, obtained from an muC103A camera sensor, from raw arthroscopic video sequences of three different cadaveric samples. Image quality is degraded by factors including motion blur (red rectangles) and additive noises (yellow rectangles). Due to lack of automatic white balance hardware, the acquired frames yield different color representations under halogen and white micro-LED illuminants.

In this work, we consider image visualization challenges of knee arthroscopy-an established MIS procedure to treat knee-joint. As part of this procedure, an imaging device (arthroscope) and surgical tools are inserted into the knee cavity. Sterile salt water (saline solution) is used to fill the knee cavity for improved navigation and visualization of this complex and dynamic joint. The imaging device captures video sequences at proximity to tissue structures (typically at around 10-mm distance)^5^ with a 30- or 70-degree field-of-view (FoV). We have developed a new class of stereo arthroscopes^5,7^ that expand the FoV to up to 110 degrees. Though an increased FoV is in general better, yet at this proximity, only small portions of the global scene context are accessible. Some example of bad image frames with noise, color cast, and blur are represented in Fig. 1.

Instances of non-uniform inter frame exposure and partly saturated pixels can further lead to visualization drawbacks in RMIS^6,7^. The most frequent type of noise observed is Gaussian noise, although other types such as speckle noise, salt-pepper noise, and Poisson noise also occur, often due to the strong backscattering produced by tissue debris^8^. Limited control on imaging device parameters and characteristics, such as exposure and aperture, unsteady hand movement (shaking), and motion caused by camera steering and maneuvering can also be a source of blurring and image artifacts. This phenomenon derives a hard problem to estimate accurate kernel adaptively and its direction, which is necessary for kernel-based deblurring methods^9–14^. Additionally, blur due to defocusing can be caused by lighting conditions, specular reflection, and improper focal settings.

In this article, the main components for the surgical scene correction such as blind denoising, blind deblurring, and automatic white balance are discussed. A deep learning-based technique has been explored to restore clear and sharp images from the original blurry, noisy, and raw RGB observations.

In this work, we demonstrate auto segmentation of visually challenging images from the knee joint using U-Net. The rationale behind use of U-Net is that it is suited to small datasets, a situation commonly experienced with medical imaging research. Moreover, skip connections and the encoder-decoder architecture of U-Net is very effective in learning semantic labels of high-level medical image features. In view of such strong sides of U-Net, in this study we have explored this Fully Convoluted Network (FCN) model as a baseline model to retrieve clean, sharp, and color corrected images considering challenging arthroscopic video sequences and lack of sharp ground truth images of knee anatomy. For instance, unlike the natural images inside the knee cavity, it is extremely difficult to collect sharp and corresponding blur video frames due to limited control and accessibility of the surgical space. This study describes a novel approach to solve IR tasks for enhanced endoscopic vision.

The main contributions of this work are:

1. Although IR tasks are well-studied in the context of natural images, in MIS very often the conventional parameterized methods are used. To the best of our knowledge, the deep learning-based frame correction method presented is novel for application to MIS. The ground truth data were partially obtained from five cadaver knee experiments. Moreover, the study shows the pros and cons of the conventional approach to address surgical scene restoration tasks.
2. Several deep learning architectures, including U-Net, have been studied in literature as either denoiser or deblurring models. In this study, we explore the viability of using the U-Net architecture to learn three frame reconstruction tasks in the context of MIS, namely: a) Denoising, b) Deblurring, c) Color correction. The model provides white color balanced, sharp and clean frames which are free from artifacts.
3. To perform three IR tasks simultaneously is a challenging problem. Coarse and fine-tuned trainings were performed in a two-stage process. We combined three loss functions into a total model loss, namely: PSNR for denoiser, PSNR and perceptual loss for color correction and structural similarity index for deblurring. Moreover, gradient loss was applied to fine tune our model to address frame blurring more accurately.
4. We verified our model outcome against all the gold standard methods. Furthermore, we evaluated model urgency to tackle higher level vision tasks, e.g. instance segmentation. Improved accuracy was observed when preprocessed frames were used using our model. Moreover, this model performed three different IR tasks in a single step, resulting in increased system performance.

## Related work

Image restoration (IR) has been well discussed in computer vision and image processing that can be expressed as follows:

1 $$I\left({x}_{i,j}\right)=G*{x}_{i,j}+\epsilon$$

where $I$ is the corrected image, ${x}_{i,j}$ is the pixel position in the two-dimensional (2D) image plane, G is a transformation matrix producing the blurring effect, and $\epsilon$ is defined as an additive white gaussian noise (AWGN) with standard deviation σ. During the IR process, blur kernels are estimated, and deconvolution operation is performed over the image. Then the noise residuals are subtracted from the resulting image.

In the context of image deblurring, several methods have been proposed^9–14,16–31^. Before the learning-based approaches, parameterized kernel methods were used to estimate motion blur^9–14,16–23^. The accuracy of these methods strongly depends on the motion kernels and on their directions. Later, several methods have been introduced that adaptively define motion kernels with the use of machine learning and deep learning approaches. However, some drawbacks of kernel-based methods have been identified^32^: (i) defining an accurate kernel is a complicated and error-prone task; (ii) methods’ accuracy is limited when a noisy environment is considered; (iii) artifacts are often introduced by these approaches when the kernel is not properly defined. Due to the limitations of the kernel-based methods, in recent years, in order to achieve non-parametric kernel-free deblurring techniques, learning-based methods have been gaining attention in the computer vision community. In particular, the method introduced in^24^ used convolutional neural networks (CNN) on multiscale image pyramids with a modified residual learning block, named ResBlock, that helps fast convergence. The method proposed in^25^ extends the capacity of CNN to solve the bicubic degradation model to restore super-resolution images from noisy input. Generative deep learning-based methods have also been successful. Deblurring is performed based on the philosophy that the generator generates a clean image from input, and the role of the discriminant is to discriminate the output of the generator which is not close to ground truth clean images. When a network gets trained, the discriminant fails to discriminate against them as the generator learns how to construct a clean image from the noisy one. The method presented in^26^ used two generative models to address the maximum posterior of the deblurring method. Similarly, in^27,28^ the authors proposed generative models with residual blocks that achieved state-of-art accuracy. In their implementation, they used two stridden convolution blocks, nine residual blocks, and two transposed convolution blocks^28^. Additionally, they used L2 perceptual loss and adversarial loss.

Denoising is also a long-standing IR task that has been well-studied previously. Traditionally, denoised images were retrieved using filter-based methods. In this context, filters can be categorized as local or non-local^34^. Local filters use a supporting window and statistical methods to interpolate the central pixel value. For non-local ones, the statistical methods are performed on several windows over the entire image for each pixel value. Gaussian^35^, non-local means^36^, and bilateral^37^ are the most common filters discussed in this section. These traditional filters produce smooth images but have a few drawbacks among which that weak edges and features tend to vanish, and consequently blurred images can be produced.

Anisotropic^38^, BM3D^39^, and total variation^40^ filters have been proposed, looking for edge-preserving denoisers. Despite the advantage of improving edge preservation, major limitations included lack of textual information, and staircase effect^34^. Moreover, in some applications, they failed to report satisfactory results^41^. In the current research, the BM3D method is considered one of the state-of-the-art methods in this area.

Apart from blurring artifacts, a major consequence of using parameterized methods is that it is not confirmed whether they can address different levels of complex noises robustly. In recent years, the robust perceptual and contextual accuracy of CNNs has promoted increased interest in the computer vision community. The method proposed in^42^ used dilated convolution with batch normalization and the ReLu activation function to extract residual noise from the noisy observations. In the method introduced in^43^, a global residual learning strategy has been followed and they named it residual dense block. Autoencoder and decoder-based architectures offer precise feature extraction and localization at each scale, which can facilitate mapping noisy back to clean images. Reference^44^ proposed DRUNet—a modified network on top of U-Net^45^ to address IR problems on half quadratic spline.

During the arthroscopic procedure, the effect of illumination can cause a slight prevalence of either the red or the blue channel, which can affect the accuracy of other vision tasks^15^. The process was defined by^15^ as follows;

2 $${I}_{sRGB}={f}_{XYZ}\to sRGB({T}_{raw}\to XYZ\, WB\, {I}_{raw})$$

where ${I}_{sRGB}$ represents image in standard RGB (sRGB) color space. Function *F* (.) maps image between CIE XYZ color space to RGB color space and transformation function *T* (.) converts image from raw RGB space to white balanced CIE XYZ color space. Mapping from raw RGB to sRGB as a part of color consistency has been explicitly discussed in many areas where illumination estimation was the key factor. Radiometric calibration and CNN have been used to address this issue^46,47^. Recently, in^15^ this mapping function has been addressed using a k-nearest neighbor strategy that retrieves a color through best matching of the nonlinear mapping function. Moreover, the authors also provided a dataset that contains 65,000 pairs of images for different camera white balance settings. In their dataset, some of the ground truth data was generated through the use of Adobe Camera Raw feature and rendered in Photoshop.

IR for endoscopic procedures has not been properly investigated yet, and the progress in this sector is limited. A scarce literature currently establishes the IR problem^48–56^ where most of the articles address specular removal, parameterized deblur, desmoke, colorization and quality assessment. Robust denoising and deblurring mechanisms in real time remains an unsolved problem which has a countless demand for robotic vision tasks such as tracking and navigating robots in the RMIS environment. More specifically, in arthroscopy, IR exhibits additional complexity considering factors such as underwater environment, lack of control on imaging devices, poor imaging conditions, lens distortion, debris presence, hazing and complex motion, which require sophisticated and robust solutions. In this article we propose a single model where raw arthroscopic images are enhanced through color correction and, irrespective of the noise level, latent clean and sharp frames are restored after simultaneous denoising and deblurring.

## Methods

Restoration of the white balanced latent clean and sharp image y from its noisy and raw sensor observation, x, is considered a mapping function:

3 $$y\to f(x,\theta )$$

where, $\theta$ are the parameters to learn during the training. In this article, this problem is considered as a regression problem.

### Model

**Figure 2:**
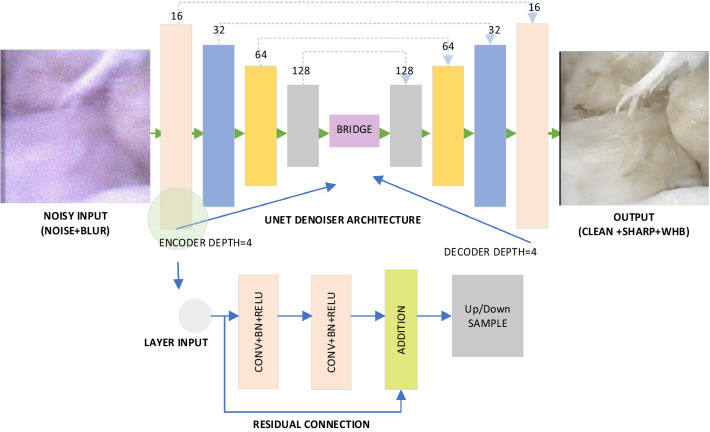
Architecture for endoscopic image restoration framework. A clean, sharp and white balanced (WHB) video frame is retrieved from its raw, noisy and blurred observation. The network depth for encoder and decoder is 4. The network uses residual connection as it is shown in the bottom image. Accumulated loss function calculated from PSNR, SSIM, perception loss and reduced mean of edge loss between noisy and clean observation.

This regression problem is addressed using a U-Net architecture, as detailed in Fig. 2.

U-Net is a well-known network architecture widely used for segmenting medical data as an end-to-end solution. In this work, the U-Net architecture is used to address color mapping and two IR tasks for MIS, namely; denoising and deblurring. Instead of classic U-Net, this implementation uses residual blocks into U-Net architecture. Following the encoder-decoder approach, the contraction path of U-Net precisely extracts features at different scales, down-sampled at each step. On the other side, the expansion path learns to localize each feature at different levels.

The residual learning^57^ strategy has several benefits across the network, it increases training and prediction accuracy even with small network depth. In U-Net architecture, spatial information loss is caused by down-sampling in the contraction path, and it has been shown that a residual learning strategy performs better over classic U-Net^58,59^. Also, recent works on IR^60^ networks such as DnCNN showed advantages from the use of residual learning strategy.

### Dataset and training

Arthroscopic video sequences have been recorded during five knee arthroscopy procedures conducted on five different cadaveric knees. During these experiments, some frames have been captured at steady camera positions. Lighting conditions were maintained consistently using manually adjusted illumination controllers. When small motion-induced blurring and defocusing were observed at some distant parts, when possible they were corrected using the methods proposed in^27,30,61^. White balances were obtained from the method introduced in^15^. Corrected color values were validated by reconstructing their reflectance and comparing them with spectrometer data as mentioned in^62^. Clean images were then artificially degraded by adding multi-level of Gaussian, Speckle, Salt and Pepper, and Poisson noise. Blur images were generated through the use of a motion blur kernel.

**Figure 3:**
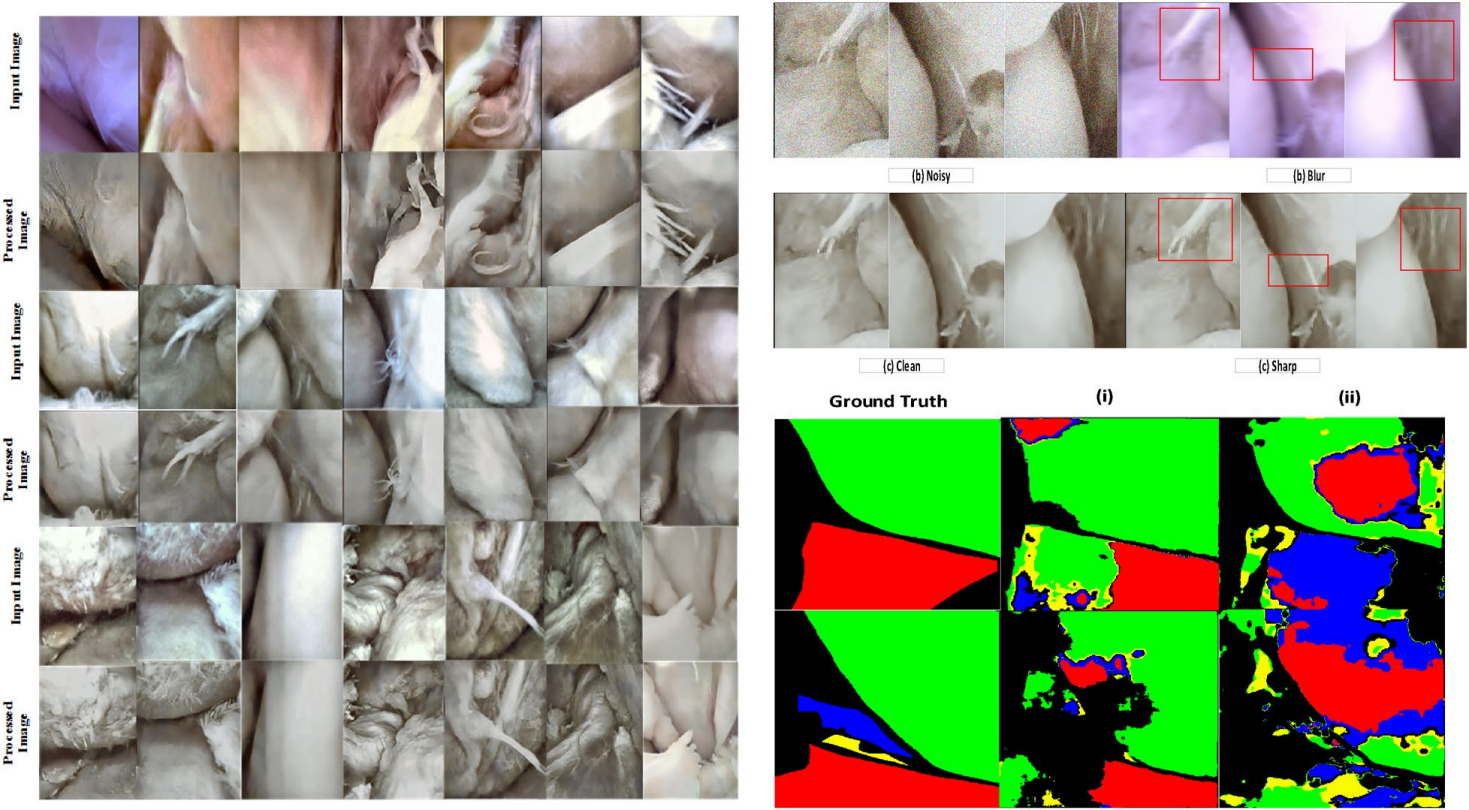
Images in the left column represents the visual representation of the real scene and the result obtained from our method. Here, the top row represents a real arthroscopic scene, and the subsequent rows represent the results. Images presented in the top right column show the outcome of IR tasks considering high-level noisy and blur data. Images at the bottom right compare ground truth segmentation with the output from or methods on arthroscopic scene segmentation. The first column represents the ground-truth label, column (i) represents segmentation results obtained from the preprocessed dataset using our method, and column (ii) represents results obtained from the same dataset without preprocessing. It is clearly showing that this framework increases the accuracy of the segmentation task.

In this work, a ResUnet strategy and Batch normalization techniques were applied. U-Net architecture consists of three basic building blocks, namely, encoder, decoder, and connecting block. Encoder block learns high-level features to its complex low-level feature representation. In this way, U-Net encoder learns coarse pixel-wise feature representation of raw images. When residual blocks are implemented on a U-Net encoder, it provides more spatial information that means more noisy spatial representations are obtained. During the convolution operation in encoder side noisy features were extracted, therefore, the model learns how to extract feature from untextured noisy and blurred frames. Similarly, on the decoder part, U-Net learns pixel-wise fine features from its high-level feature’s representations, for instance, blur weak edges to its sharp representation. It subsequently preserves contextual information thus producing a clean and sharp image in an end-to-end fashion. Batch normalization is widely recognized for faster training when input distributions are different, known as an internal covariate shift. Reference^42,60^ methods received benefits with the use of batch normalization to learn noisy residual images. Noise such as gaussian and others can have different statistical distributions around the arthroscopic sequences. Moreover, blur can occur at different levels from multiple motions, a well-observed occurrence in underwater MIS. It is understood that these motions can exhibit blur effects at different directions in images, even a single pixel can experience several motions having different motion directions. To incorporate level independent noises including blur effect which means diverse input distributions, batch normalization strategy has been followed. Along with these we used 400 image samples from^33^ and the whole dataset, as shown in Fig. 3, was split into three categories: (i) clean images; (ii) blurred images, (iii) noisy and blurred images.

U-net architecture also learns to localize its features from local scope to global. To facilitate this in the context of arthroscopy, we also used synthetically rendered arthroscopic video sequences using 3D graphics software-Blender. To do so, we used 91 samples for each of the five attenuation types which are noisy, blur, speckle, salt pepper, and poisson. For validation we used one third of natural and rendered images described above. Along with these images, the training has been performed on 4500 cadaver knee images. 1500 images were used as a validation dataset which contains both natural, rendered, and cadaveric video frames. During the test, we used a total of 6803 arthroscopic video frames from all five cadaver samples.

During the training structural similarity index (SSIM), peak signal to noise ratio (PSNR), perception loss- L2norm, and loss of edges between noisy and clean observations have been evaluated individually. It has been found that with the use of accumulated loss function of SSIM, PSNR and L2 norm the network converged smoothly and obtained better validation and test accuracy. The total loss is defined as,

4 $${Loss}_{total}=\sum ({L}_{SSIM}+{L}_{PSNR}+{L}_{2})$$

Loss ${L}_{PSNR}$ and ${L}_{2}$ norms are used to define the network learning strategy to reconstruct a clean image from its noisy observation as well as the color mapping function. PSNR is defined as follows^63^;

5 $$PSNR= 10\mathrm{*log}10\left(\frac{{max}^{2}}{MSE}\right)$$

where,

6 $$MSE= \frac{1}{M*N*O}\sum_{x=1}^{M}\sum_{y=1}^{N}\sum_{z=1}^{O}\left[{({I}_{\left(x,y,z\right)}-{I}_{(x,y,z)}^{^{\prime}})}^{2}\right]$$

In Eq. (5), max represents the highest value of a grayscale image. Similarly, SSIM and the difference of edges between a noisy and blur image are used for deblurring during the fine-tuned training stage. In this strategy, the network learns sharp edges and features from its coarse blur representations.

Learning noises is a comparatively simpler task than deblurring. Moreover, deblurring under noisy conditions is a relatively more complex task than straightforward deblurring. We followed two-stage training procedures, (i) coarse training, and (ii) fine-tuned training. The network outperforms, when it is trained with all noisy and blur observations for coarse training and then fine-tuned training is done over the blur dataset.

The arthroscopic dataset contains noisy observations, named as real and its corresponding clean images act as ground truth data. The real data set contains raw observation of arthroscopic scenes which are compromised to noise and blur. Moreover, these frames are not white balanced and provide raw RGB color. It is worthwhile to mention that, many frames perceptually exhibit small contextual information due to lighting conditions that are not uniform inside the knee cavity, therefore, the frame contains both saturation and underexposed image parts. Additive white gaussian noise (AWGN) is added to the raw input frames with standard deviation^25^. To simulate debris, haze, and random backscattering noise like speckle, salt-pepper, and Poisson are added to the real video frames. Additionally, to achieve several levels of blurring effects, both real and raw images are convolved with blur kernels. Training phases used Adam optimizer with learning rate 1e-4. It takes 0.024 s to process each frame with the use of the Nvidia Tesla -P100 GPU.

## Results

To compare IR results, the state-of-the-art algorithms, including, Gaussian^35^, non-local mean filter^36^, Bilateral filter^37^, BM3D^39^, For deblurring BM3D-deblur^39^, Bayesian-based iterative^64^, unsupervised wiener^65^, l0 gradient prior^66^, Total variation deconvolution^67^, natural image statistics^68^, deblurring under high noise levels^69^ and deep learning based method, deep CNN denoiser prior^42^, Deblur GAN^28^, Scale-recurrent network^30^ are evaluated. In all the tables font bold represents highest score, bold and italics represent second highest score, and italics only represents third highest score.

Table 1 represents the outcome of our model and others at different noise levels. To do so, Gaussian additive noises were added to the input arthroscopic frames. Evaluation were modelled using both classic state-of-the-art conventional methods and recent learning-based methods. To denoise endoscopic scenes classical mathematical methods such as Bilateral filter, BM3D are widely used, however, learning based method like IRCN has proved an effective way to denoise frames^37,39,42^. From the Table 1, it can be seen that to address various levels of noises, in medical domain U-Net model constantly achieved high accuracy (> 92% up to noise level sigma = 40) while it simultaneously performs three IR tasks. Although compare to learning based method like IRCN and ours, the BM3D model achieved high average accuracy, it was able to perform denoise task only. It is worthwhile to note that, when BM3D model jointly perform denoise and deblur it achieved lower accuracy compare to BM3D denoiser. It confirms that, combinedly perform denoise, deblur, and color correction is a challenging task where U-Net and the proposed workflow has significant potential to solve this problem. With real world arthroscopic dataset (without artificial noises) it achieved 94% SSIM index.

**Table 1:** Gaussian de-noise.

Method	σ = 10		σ = 20		σ = 30		σ = 40		σ = 50		σ = 60	
	SSIM	PSNR	SSIM	PSNR	SSIM	PSNR	SSIM	PSNR	SSIM	PSNR	SSIM	PSNR
GAS^35^	0.936	31.16	0.84	30.26	0.726	29.04	0.626	27.7	0.536	26.36	0.465	25.09
NLM^36^	0.942	38.7	0.91	35.7	0.771	29.80	0.512	24.03	0.310	20.13	0.19	17.0
BILT^37^	**0.960**	41.36	0.89	37.12	0.670	31.48	0.410	26.7	0.245	23.19	0.157	20.6
BM3D^39^	0.936	30.8	0.932	30.61	***0.925***	30.13	***0.914***	29.3	**0.899**	28.29	**0.899**	29.5
BM3D DEBLUR^39^	0.898	22.59	0.896	22.93	0.893	31.41	0.885	23.5	0.870	23.8	0.844	24.03
IRCN^42^	***0.956***	40.0	**0.956**	39.0	0.819	33.29	0.428	25.92	0.231	22.14	0.141	19.6
OUR	*0.93*	34.1	***0.94***	35.0	**0.93**	33.9	**0.92**	31.9	***0.82***	29.2	***0.61***	25.82

Table 2 shows the evaluation of speckle, salt pepper, poisson noises which simulate debris in arthroscopic video frames. Similar to the previous discussion performing denoise only BM3D achieved highest SSIM index (86%) where our model (denoise, deblur, and color correction) achieved 84% SSIM. However, our model achieved higher PSNR index compare to BM3D models. Learning based denoiser and deblurring model such as IRCN^42^, SRN^30^ and GAN^28^ achieved relatively low accuracy compare to our and BMD model.

**Table 2:** Speckle, salt pepper, Poisson noises.

Method	SSIM	PSNR
GAS^35^	0.825073	26.783526
NLM^36^	0.803788	30.834656
BILT^37^	0.792327	31.219724
AISO^38^	0.730226	25.649356
BM3D^39^	**0.865542**	26.563407
BM3D DEBLUR^39^	0.825373	20.884970
IRCN^42^	*0.832018*	**31.624574**
GAN^28^	0.736981	24.844043
SRN^30^	0.756599	30.071061
LCY^64^	0.765477	32.147631
OUR	***0.84***	***30.63***

Table 3 shows the evaluation of deblurring techniques. Our proposed model achieved highest SSIM and PSNR index compared to other learning based and classic computational deblurring methods. Table 4 shows the ability of IR models to perform three rudimentary tasks namely denoise, deblur, and color corrections. So far, to our best knowledge, our model has been evaluated first in literature to achieve these three IR tasks in a single shot manner. Our results also justify that, rather than adapting IR generic models such as for natural images, in medical domain it is necessary to have domain specific training. It is due to endoscopic images are challenging, and their appearances fundamentally constrained by low textures, body fluid, illuminations, and artifacts.

**Table 3:** Deblur.

Method	SSIM	PSNR
BM3D DEBLUR^39^	0.85	21.2
IRCN^42^	0.89	19.8
GAN^28^	0.85	25.2
SRN^30^	***0.92***	27.5
LCY^64^	*0.90*	27.5
WIN^65^	0.80	32.2
10GR^66^	0.82	29.0
TV^67^	0.83	24.5
NI^68^	0.87	21.6
HN^69^	0.76	21.1
OUR	**0.94**	**37**

**Table 4:** Denoise, deblur, and color correction.

Method	Denoise	Deblur	Color correction
GAS^35^			
NLM^36^			
BILT^37^			
AISO^38^			
BMD^39^			
BMD^39^ DEBLUR			
IRCN^42^			
GAN^28^			
SRN^30^			
LCY^64^			
WIN^65^			
10GR^66^			
TV^67^			
NI^68^			
HN^69^			
OUR			

In this table,  means the ability of a method to perform IR task and  means the method cannot be applied to perform that IR task.

**Figure 4:**
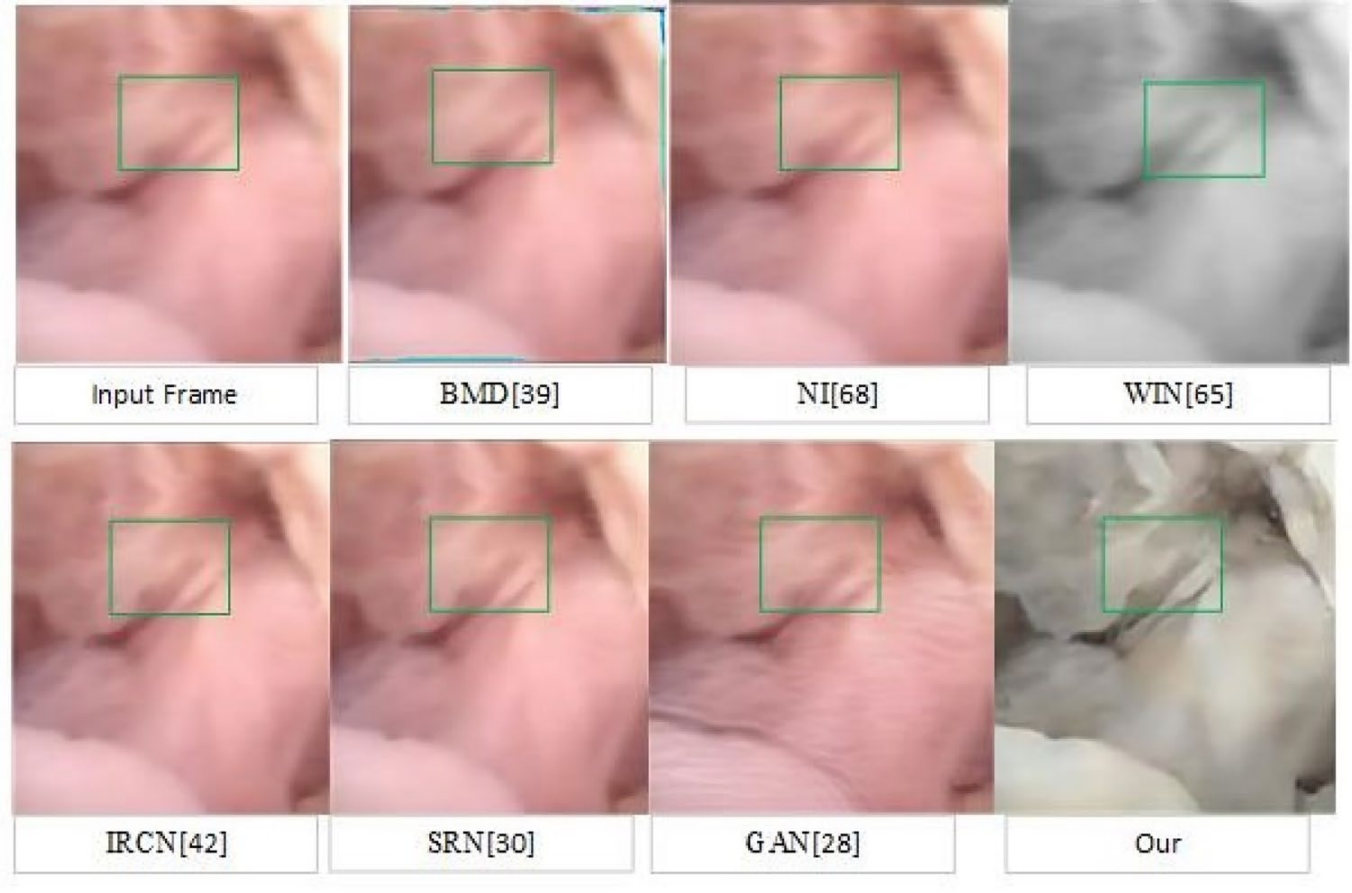
Visual comparison of the deblurred frame obtained from traditional, deep learning, and our method. As one can see, our method retrieved sharp texture and white balanced frame.

**Figure 5:**
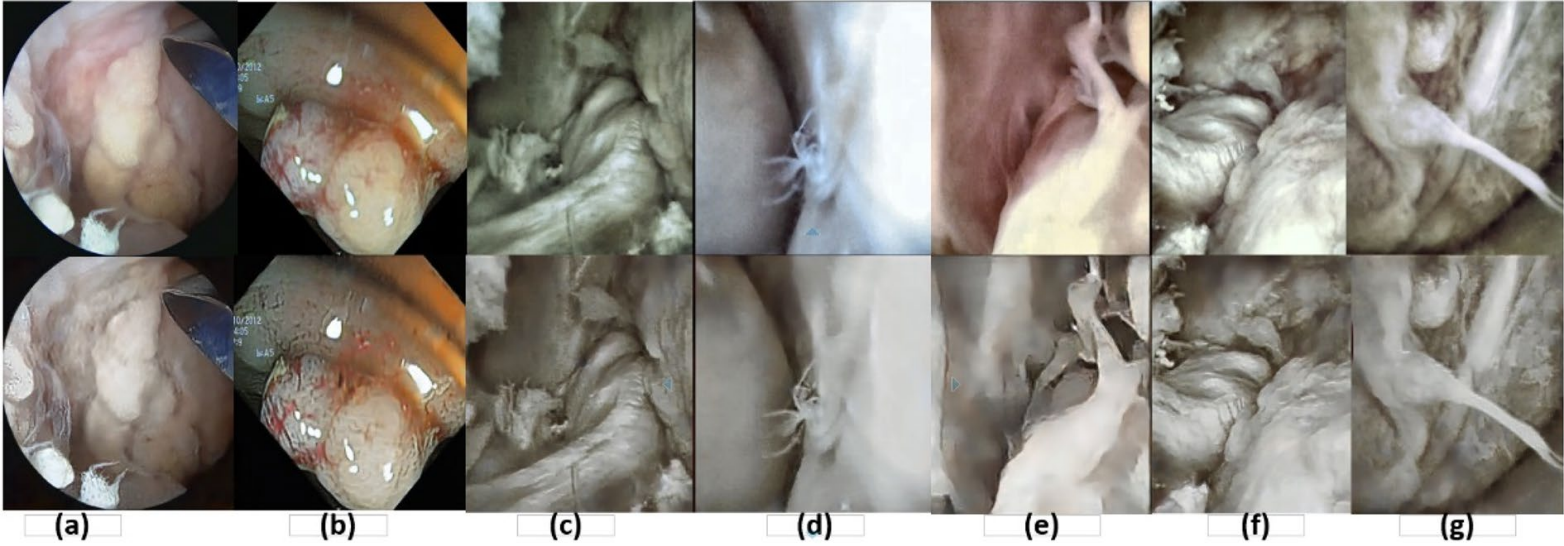
Presentation of original (upper row) and pre-processed frames (second row). (**a**) Arthroscopic frame taken from Stryker camera and not used during training. (**b**) The endoscopic frame of the gastrointestinal tract which were not used during training. In both images are enhanced through the retrieval of textures (edges). Similarly, (**c–g**) represents arthroscopic frames under different illumination. In all cases, different levels of noises and blur exist which were corrected by our method. Deblurred and denoised frames show enhanced texture information.

Perceptual representations are presented in Figs. 3, 4, and 5. To demonstrate the impact of our method on high-level vision tasks, arthroscopic scene segmentation is performed. The same neural network used by method^5^ is trained for this task using both raw and preprocessed data using this method. On the same test set, the accuracy improvement for Femur, Anterior Cruciate Ligament (ACL), Tibia, Meniscus are 2.6%, 2%, 6.3%, and 7% (Fig. 3).

## Conclusion

Image restoration is a critical part of high-level vision tasks such as stereo matching, monocular depth, and segmentations in the context of knee arthroscopy^70–75^. It is confirmed from the obtained results that, our proposed framework restored clean and enhanced frames consisting of more textual information. Moreover, our method can restore frame details with higher-order noise levels. The framework uses established encoder-decoder like convolutional neural network architecture -U-Net with a strategy like Residual learning and batch normalization to speed up the training phase. The resultant network delivers highest accuracy when perceptual loss, PSNR, SSIM, and edge difference loss are summed up in a two-stage training.
